# Quality of life during chemotherapy, hormonotherapy or antiHER2 therapy of patients with advanced, metastatic breast cancer in clinical practice

**DOI:** 10.1186/s12955-020-01389-x

**Published:** 2020-05-12

**Authors:** Krzysztof Adamowicz, Zuzanna Baczkowska-Waliszewska

**Affiliations:** 1 Pomeranian Oncology Center, Gdynia, Poland; 2 Department of Oncology, Pomeranian Hospitals in Wejherowo, Powstania Styczniowego 1, Gdynia, Poland; 3 7th Naval Hospital, Gdansk, Poland

**Keywords:** Advanced breast cancer, Quality of life, Chemotherapy, Hormonotherapy, Treatment outcomes

## Abstract

**Introduction:**

Breast cancer is one of the most important health problems in the world. In recent years, this cancer has achieved a reduction in mortality, which is attributed to the introduction of mass screening and greater efficacy of post-operative treatment. Many patients with breast cancer have indications only for palliative therapy, but the impact of these methods on the quality of life of patients remains a subject of controversy. It remains unknown whether the progress in improving the quality of life in clinical trials also applies to patients treated as part of daily clinical practice. Data on the results of the impact of conducted therapies on the quality of life outside of clinical trials are scarce.

**Methods:**

The results of palliative chemotherapy and first-line hormonotherapy in 351 patients with advanced, metastatic breast cancer treated in the period from January 2010 to December 2016 in two centres were analysed.

**Results:**

The average age of patients was 62 ± 9.8 years; 139 patients received chemotherapy, 91 - therapy containing trastuzumab, and 121 - hormone therapy. A partial response was obtained in 111 patients (32%), stabilization in 150 (43%), and in 90 patients (26%) progression. Median survival time in the whole group of patients was 36 months. Chemotherapy compared to trastuzumab and hormonotherapy was associated with greater total toxicity (*p* = 0.03). There was a significant relationship between the type of therapy (hormonotherapy, chemotherapy, targeted therapy) and the general average quality of women’s life measured with the EORC-QLQ-C30 questionnaire. In addition, a statistically significant difference was found in some somatic complaints (the scale of QLQ-BR23 symptoms) depending on the type of therapy performed. The lowest intensity of complaints was reported by patients during hormonotherapy, then during targeted therapy, and the largest during chemotherapy.

**Conclusions:**

There is no effect of chemotherapy on the overall quality of life. Hormone therapy and trastuzumab therapy improved the quality of life of the treated patients in clinical practice.

## Introduction

Breast cancer is one of the most serious health problems worldwide. Every year, about 1,500,000 new cases and 400,000 deaths due to this disease are recorded [1]. Breast cancer is the most common malignancy affecting women in Poland. In recent years, according to the National Cancer Registry, the number of cases per year has exceeded 16,500. In Poland, breast cancer has been the second, after lung cancer, cause of deaths related to malignant tumors among women (the number of deaths - about 5500 per year) [2]. In the last decade breast cancer mortality in the European Union has decreased because of mammography screening introduction, as well as the increasing effectiveness of adjuvant therapy. Currently, most early breast cancer patients receive breast conserving therapy with the use of radiotherapy. Many of them also require pre-operative or adjuvant systemic treatment (chemotherapy, hormone therapy, trastuzumab). A particularly poor prognosis concerns patients with multiorgan metastases who do not qualify for surgical treatment. In the last decade, the improvement in the median total survival in this group of patients was achieved due to modifications of previously used treatment regimes, appropriate sequencing of treatment methods, but primarily as a result of HER2 receptor-directed therapy. A better understanding of breast cancer biology has led to the development of molecular-targeted therapy - monoclonal antibodies: trastuzumab and pertuzumab and the tyrosine kinase inhibitor of EGFR and HER2 receptors - lapatinib. In addition to indicators such as total survival or disease-free survival, an important factor determining the introduction of a new clinical standard is also the toxicity of treatment and the quality of life of the treated patients. Treatment toxicity is routinely assessed in clinical trials, while quality of life is assessed less frequently.

However, prospective studies involving new drugs usually include selected groups of patients, in good general condition, with no significant comorbidities and good organ function. On the other hand, in everyday clinical practice many patients are elderly, in poor general condition and have numerous comorbidities. It is therefore relevant whether the results presented in clinical trials can be related to those achieved in daily clinical practice. Data on the results of treatment of patients with advanced, metastatic breast cancer in everyday clinical practice are scarce. What is more, the disease itself, as well as its social and psychological consequences may significantly affect the current quality of life of patients [3–7]. However, the majority of studies on the quality of life of breast cancer patients concern the aspect of aesthetics and symbolism implied by breast amputation [3–7]. An important aspect of those studies is the analysis of social, occupational and sexual life as well as the body image both in the subjective dimension and the social perception of the surveyed women [4–7]. Bearing the above data in mind, the authors of this article undertook the task of prospective analysis of quality of life, body image, adverse effects of treatment, sexual satisfaction and prospects in a group of women diagnosed with advanced, metastatic breast cancer depending on the treatment method used. In this instance, however, the study concerns palliative patients. The authors` study, involving a large group of patients undergoing palliative chemotherapy, hormone therapy and anti-HER2 therapy as part of daily clinical practice, is an attempt to assess the actual effectiveness of treatment in this population, with focus on the quality of life of patients treated.

## Methods

The subject of the study was 351 patients with advanced, metastatic breast cancer who from January 2010 to December 2016 received palliative chemotherapy, hormone therapy or anti-HER2 first line therapy at the Specialist Hospital in Wejherowo and at the Regional Centre of Oncology in Gdansk. The group consisted of patients with primary or secondary dissemination who were disqualified from metastases resection. The study was prospective.

Age, sex, education, smoking, alcohol consumption, family history, height, weight, pain intensity, method and treatment regimen, were the subject of analysis. Persons defined as current smokers were those who during the last 12 months have smoked at least one cigarette daily. Regarded as former smokers where those who have not smoked in 12 months, and non-smokers those who have never previously habitually smoked. A positive family history of cancer was defined as breast and ovarian cancer in first and / or second-degree relatives. These data were obtained from patients based on the interview. Body weight and height were recorded for all patients at the start of treatment. The response was assessed using the RECIST 1.1 scale, based on subsequent CT studies. This evaluation, regardless of the reports made, was carried out centrally.

Data related to treatment toxicity was analysed in accordance with WHO adverse drug reaction classification as observed on the first planned day of next chemotherapy cycle, regardless of its regimen. Hematologic toxicity was assessed based on actual laboratory results, performed on the day of planned chemotherapy administration. Remaining adverse reactions were analysed using the medical records kept by the attending physician.

Quality of life was evaluated with the use of EORTC QLQ-C30 form [22]. Questionnaires in the Polish language version were filled out by patients during the last week prior to beginning of treatment and the first week after completion of treatment.

Basic descriptive statistics were used for the result analysis. Arithmetic average was equated with the use of t-Student test in the case of two variables or ANOVA analysis for more than two variables. Logistic regression (age, sex, family history, alcohol consumption, smoking status, intravenous vitamin C administration) was employed to determine the relation between the applied treatment regimen and response to treatment. *P* value of less than 0.05 was considered as statistically significant. Additionally, analysis of regression and variable correlation was performed. Enough sample size was determined.

The influence of individual factors on overall survival was evaluated through Cox proportional hazard regression with credibility quotient testing. Toxicity and quality of life of patients receiving treatment regimens was determined with the application of logistic regression, considering age, sex, family history, alcohol consumption, smoking status and intravenous vitamin C administration. In the quality of life assessment, specific instructions provided by EORTC were considered. Statistical analysis was performed with the use of Microsoft Excel 2016 and PQSTAT programme, version1.4. The study was approved by heads of both units and the Bioethical Commission at the District Chamber of Physicians in Gdansk.

The condition for obtaining it was to present the database with complete anonymity of individual patients’ data.

## Results

Clinical characteristics are presented in Table 1. The mean age of the studied group was 62 ± 9.8 years, 29% were patients over 70 years old, 100% were women, and 42% patients with primarily disseminated cancer, 80% women had organ metastases (extraosseous and extranodal). The majority of patients were married women (78%), residing mainly in the city (66%). Half of them had secondary education. Among respondents, about 60% were people who assessed their financial situation as good and living with their families (about 96%).

**Table 1:** Clinical characteristics of patients

Characteristic	Number
Performance status	
0	168 (48%)
1	168 (48%)
2	15 (4%)
Primary location	
right breast	168 (48%)
left breast	183 (52%)
Age (years)	
31–40	26 (7%)
41–50	63 (18%)
51–60	70 (20%)
61–70	91 (26%)
71–80	91 (26%)
81–90	10 (3%)
Family history of neoplastic disease	
yes	53 (15%)
no	298 (85%)
Place of residence	
city	232 (66%)
rural area	119 (34%)
Smoking status	
yes	133 (38%)
no	218 (62%)
Education	
lower than secondary	88 (25%)
secondary	186 (53%)
higher	77 (22%)
Disease dissemination	
primary	147 (42%)
secondary	204 (58%)
Chemotherapy regimen	
chemotherapy	139 (40%)
hormone therapy	121 (34%)
anti-HER2 therapy	91 (26%)

139 patients received chemotherapy, 91 – regimen that included trastuzumab therapy (at the time pertuzumab in Poland was not reimbursed, and lapatinib reimbursed in the second line of therapy), and 121 – hormone therapy. In patients over 70 years, hormone therapy was more frequently used, even in the case of organ metastases (*p* < 0.01). In patients with a poor general condition, hormone therapy was more often used (*p* < 0.01). No relationship was found between age and general condition of patients (*p* = 0.13).

A partial response was obtained in 111 patients (32%), stabilization in 150 (43%), and in 90 patients (26%) progression was noted. Median survival time in the whole group of patients was 36 months. The median survival time for targeted therapy, chemotherapy and hormone therapy was 38 months, 35 months and 34 months, respectively. In the parametric and multivariate analysis, the response to treatment was not related to the general condition (*p* = 0.77), age (*p* = 0.45), education (*p* = 0.92), the type of disease generalization (primary vs. secondary) (*p* = 0.96) or smoking (*p* = 0.55). A relationship was found between the response to treatment and the type of therapy used (targeted therapy vs other methods *p* < 0.01, chemotherapy vs. hormone therapy *p* = 0.17).

Of the 351 patients included in the study, 47 were still alive at the time of the analysis. The median survival time in the whole group was 36 months. In multivariate analysis, the survival status was significantly affected by the performance status and response to treatment (Table 2).

**Table 2:** The survival time, depending on the demographic and clinical factors (multivariate analysis)

Variable	Risk ratio (95%Cl)	p
**ECOG performance status**	**1,54 (1,21-1,98)**	**0,01**
Type of therapy	0,82 (0,61-1,02)	0,08
Age	0,91 (0,74-1,16)	0,11
Education	1,01 (0,81-1,21)	0,94
Place of residence	1,10 (0,78-1,42)	0,71
Treatment centre	1,05 (0,75-1,45)	0,88
Smoking status	0,91 (0,80-1,02)	0,19
**Response to treatment**	**0,64 (0,44-0,83)**	**< 0,001**

The most common adverse reactions were haematological toxicity, including neutropenia, thrombocytopenia and anaemia, as well as peripheral neuropathy, vomiting and diarrhoea. Less frequent side effects were: infections, hair loss, fatigue and constipation. All adverse reactions were more common among patients treated with chemotherapy. Overall toxicity was not related to the general condition (*p* = 0.34), education (*p* = 0.43), place of residence (*p* = 0.55), family history (*p* = 0.31), smoking (*p* = 0.07), primary or secondary disease generalization (*p* = 0.81), diabetes (*p* = 0.09), hypertension (*p* = 0.26) and response to treatment (*p* = 0.69). Overall toxicity depended on the type of therapy (chemotherapy versus hormone therapy, *p* = 0.02) and age (below or over 70 years, *p* = 0.003). The risk of febrile neutropenia and peripheral neuropathy was higher in patients with concomitant diabetes (*p* = 0.01 and *p* = 0.03 respectively). The incidence of diarrhea, neutropenia, thrombocytopenia, anemia and the frequency of hospitalization was higher among patients treated with chemotherapy. The frequency of dizziness and hot flashes was higher in the case of hormone therapy, while peripheral neuropathy and lower limb edema occurred more frequently in patients receiving trastuzumab. None of the patients during treatment died due to treatment toxicity. Owing to treatment toxicity chemotherapy was postponed in 21.1% administrations. Neutropenia was the cause of 80.3% deferrals, including neutropenic fever 6.3%, diarrhea (5.5%), anemia (3.5%), thrombocytopenia (2.9%) and neuropathy (2.9%). The most common causes of dose reduction, which affected 5% of patients, were peripheral neuropathy and febrile neutropenia. The severity of pain before and after treatment did not differ significantly (*p* = 0.33) and was not related to the response to treatment (*p* = 0.19).

### Quality of life

The quality of life questionnaire was completed by all patients. All patients completed the form both before and after the treatment. About 10% of patients, however, missed completing the survey during the therapy. During chemotherapy and anti-HER2 therapy, the questionnaire was completed on the day the therapy was administered. During the hormone therapy the questionnaire was filled out every 12 weeks. Considering all treatment methods, the overall quality of life after treatment did not differ from the quality of life before treatment. In relation to the whole group, only an increased frequency of diarrhoea, nausea, vomiting and financial problems was observed during treatment (Table 3). Pain intensity (according to the Visual Analog Scale) before and after the treatment did not differ significantly (*p* = 0.14) and was not related to the response to treatment (*p* = 0.19). The quality of life before treatment was not related to age, education, place of residence and smoking. A relationship was found between the general quality of life and the performance status (0 vs > 0) and the presence of organ metastases (advanced stage of the disease). After the treatment was completed, the quality of life depended on the response to the treatment and the treatment method used.

**Table 3:** QLQ-C30 questionnaire results

QLQ-C30	Before treatment			After treatment		
	Average	Standard deviation	Median	Average	Standard deviation	Median
Performance scales^a^						
Physical performance	71,77	20,66	72,13	68,27	21,22	69,33
Role fulfilment (work/family)	63,87	29,95	63,67	55,22	29,33	57,25
Emotional functioning	68,44	25,70	68,97	67,66	24,58	68,33
Intellectual performance	72,71	26,18	70,97	70,71	26,25	70,66
Social functioning	73,23	27,94	76,33	74,25	17,99	76,66
Overall quality of life (Qol)	60,92	23,71	58,33	58,20	19,88	58,66
Symptom scales^b^						
Fatigue	48,33	20,26	44,63	49,11	19,98	50,15
**Nausea and vomiting**	**6,87**	**8,32**	**6,67**	**24,33**	**10,25**	**23,99**
Pain	29,88	19,33	26,87	28,55	18,41	27,50
Dyspnoea	23,39	13,35	24,10	22,11	15,42	23,10
Sleep disorders	36,33	22,11	36,66	34,99	19,87	33,33
Appetite loss	19,24	12,77	19,01	20,66	11,88	20,30
Constipation	20,77	17,98	23,33	19,54	16,66	18,80
**Diarrheal**	**9,90**	**11,01**	**10,40**	**25,55**	**18,87**	**27,05**
**Financial problems**	**29,67**	**29,17**	**31,33**	**40,40**	**30,58**	**44,20**

The significant differences in bold

^a^ A higher score indicates a better level of functioning and quality of life; min. 0, max. 100

^b^ A higher value indicates more severe symptoms; min. 0, max. 100

After 6 months of therapy, most respondents (over 66%) stated that they did not suffer fatigue, 23% of respondents felt a clear lack of energy, and about 11% of respondents remained undecided. In this respect, there were no differences in individual treatment methods. Patients most often complained of weakness (over 75% of respondents, 80% respectively for chemotherapy, 60% for hormone therapy and 75% for targeted therapy). A large number of patients (41%) reported nausea and vomiting, 60% respectively for chemotherapy, 20% for hormone therapy and 55% for targeted therapy. Only slightly less frequently, the respondents noted appetite loss and constipation (37 and 35% respectively, irrelevant of treatment method). Among daily life limitations resulting from the treatment, the majority of patients at working age (over 58%) indicated the lack of possibility to continue work. In turn, in the whole group of patients, inability to perform everyday activities was often reported (67%). Neither restrictions noted by patients were related to the methods of therapy. About 10% of patients spent most of their time in bed, despite their performance status being at most ECOG = 2. The vast majority of patients (88%) received emotional support from their families, while 1% of patients reported that they were deprived of any support from their relatives. According to approximately 50% of respondents, the diagnosis and treatment of cancer did not significantly affect their family life, however, in the case of nearly 50% of patients, the disease had negative effects on their closest family (61% for chemotherapy, 37% for hormone therapy and 51% for targeted therapy). For the majority of respondents (51%) the diagnosis of breast cancer and related treatment did not disturb their social life. However, over 40% of respondents reported that the disease limited their social activity (70% for chemotherapy, 23% for hormone therapy and 50% for targeted therapy, respectively). Only 33% of patients did not report feeling sad or apathetic, however, up to 60% of patients had such feelings (irrelevant of method of therapy). In addition, nearly 10% of patients described their health as good, and the vast majority of respondents (88%) - as poor.

Among the surveyed, as much as 72% were afraid of treatment before its start, (respectively 96% for chemotherapy, 44% for hormone therapy and 95% for targeted therapy). After a period of 6 months of therapy, only 23% of respondents were anxious about further treatment- respectively, 33% for chemotherapy, 5% for hormone therapy and 30% for targeted therapy.

A number of patients (45%) were anxious about changes in their appearance (100% for chemotherapy, 11% for hormone therapy and 44% for targeted therapy). Patients were informed and educated about their disease mainly by doctors (over 90%), to a small extent this information was obtained from nurses, family, as well as from books and brochures, the Internet or from other people suffering from the same disease. In nearly half of the patients (48%) the diagnosis of cancer and its treatment resulted in a deterioration of their financial situation. Half of the surveyed patients with breast cancer did not note the negative impact of cancer on their finances.

In the studied population, the average overall quality of life before treatment was 60.92, and after treatment 58.20. The results of the EORTC QLQ-C30 and QLQ-BR23 questionnaires completed before and after treatment are presented in Tables 3 and 4.

**Table 4:** QLQ-BR23 questionnaire results

QLQ-BR-23	Before treatment			After treatment		
	Average	Standard deviation	Median	Average	Standard deviation	Median
Performance scales^a^						
Body image	64,71	31,55	65,60	62,15	28,78	63,33
Sexual performance	12,80	17,09	13,33	15,20	16,22	16,66
Sexual satisfaction	44,42	21,96	40,25	40,98	26,15	42,58
Future prospects	31,76	32,15	36,25	33,65	24,44	35,78
Symptom scales^b^						
**Adverse reactions to treatment**	**11,93**	**18,20**	**13,66**	**34,58**	**19,25**	**35,55**
Breast- related symptoms	21,93	21,68	17,98	19,80	17,77	20,17
Arm- related symptoms	21,98	12,99	22,55	20,55	13,67	22,41
**Hair loss**	**14,84**	**10,30**	**13,33**	**35,41**	**9,25**	**36,66**

The significant differences in bold

^a^ A higher score indicates a better level of functioning and quality of life; min. 0, max. 100

^b^ A higher value indicates more severe symptoms; min. 0, max. 100

A significant relationship was established between the type of therapy (hormone therapy, chemotherapy, targeted therapy) and the general average quality of life assessed with the EORC-QLQ-C30 questionnaire. In addition, a statistically significant difference was found for some somatic complaints (QLQ-BR23 scale for symptoms) depending on the therapy method. The lowest symptom severity was reported by patients treated respectively with hormone therapy, targeted therapy, and the largest during chemotherapy. The difference noted was statistically significant (*p* = 0.018). (Table 5)

**Table 5:** Quality of life according to demographic and clinical factors on (multivariate analysis)

Variable	Risk ratio (95%Cl)	p
ECOG performance status	1,35 (1,15-1,55)	**0,01**
Target therapy addition	0,91 (0,75-1,07)	0,19
Age	1,03 (0,90-1,16)	0,22
Education	1,05 (0,90-1,20)	0,37
Smoking status	0,95 (0,88-1,03)	0,09
Response to treatment	0,78 (0,68-0,88)	**< 0,01**
Chemotherapy/hormone therapy	1,20 (1,10-1,31)	**< 0,01**

A relationship was found between the type of therapy, cognitive performance and the severity of systemic treatment side effects. Women undergoing hormone therapy demonstrated better cognitive performance and at the same time reported less severe systemic side effects of treatment than women treated with chemotherapy. These findings were statistically significant (Table 6).

**Table 6:** Cognitive performance and side effects of systemic treatment in relation to surgery type, based on QLQ-C30 and QLQ-BR23 questionnaire results

QLQ-C30	Type of therapy	Average	Standard deviation	Median	U Manna–Whitney test
Performance scales*					
Cognitive performance	chemotherapy	64,45	23,11	63,67	*p* = 0,031
	Hormone therapy	81,16	21,82	83,67	
QLQ-BR-23	Type of therapy	Average	Standard deviation	Median	U Manna–Whitney test
Symptom scales**					
Side effects of systemic treatment	chemotherapy	58,97	11,73	59,00	*p* = 0,003
	Hormone therapy	28,33	16,11	29,34	

For all treatment methods, regardless of the use of vitamin C infusions, the overall quality of life measured by QLQ-C30 and BR23 following treatment did not differ from the quality of life before treatment. Among all patients, 92 (26%) reported regular intravenous infusions of vitamin C regardless of oncological treatment. In the group using intravenous vitamin C infusions, there were significantly more patients with lower education and a strong family history of breast and/or ovarian cancer. Apart from that, there were no differences in the demographic and clinical factors analysed between the groups. After treatment, the quality of life in both groups was associated with the response to treatment and the treatment method used. During treatment, diarrhoea, nausea and vomiting were more common in both compared groups. In addition, increased financial problems were more frequent in the group receiving vitamin C. Patients in the group receiving vitamin C had financial problems more often than in the group not receiving infusions (56 and 48%, respectively; *p* < 0.01).

Additionally, in multiple regression analysis, a significant positive correlation between the “emotional performance” variable (questions 21–24 QLQ-C30) and the future prospects assessment (*p* < 0.01) was demonstrated. In addition, a relationship between the type of treatment and cognitive functioning was demonstrated. Women undergoing hormone therapy were characterised by significantly less cognitive impairment than patients receiving chemotherapy. In the group receiving the infusions cognitive functions deteriorated regardless of the treatment method used, however in the group not receiving vitamin C infusions, patients undergoing hormone therapy had significantly less cognitive impairment than those undergoing chemotherapy (*p* < 0.01).

## Discussion

Radical surgery in the context of cultural perception of the breast affects the quality of life of the treated patients. Consequently, both breast cancer and its treatment may significantly affect not only the woman’s somatic health, but also the quality and style of her life, sexuality and body image [8]. Removing the breast and its functionally related parts of the lymphatic system may cause numerous anatomic-physiological disorders, which may result in everyday function restrictions. Data on the quality of life was met with a lot of interest and influenced guidelines concerning limiting the extent of surgery in radical treatment [9–11]. Numerous authors also agreed that the removal of a woman’s breast has a strong impact on her emotional, social and family life [8, 12]. Despite advances in the diagnosis and treatment of breast cancer, breast cancer patients continue to experience problems in the various areas that contribute to their subjective sense of quality of life. Therefore, the study of the quality of life of palliative breast cancer patients and subsequent attempts to optimize the therapeutic process seem to be of current relevance.

In the presented group of patients, the median survival time was 36 months. The results of treatment, determined by survival time and response to treatment, were slightly worse compared to the results currently obtained in prospective phase III trials. The results of the general quality of women’s life obtained by the authors, which amount to 60.92, do not differ significantly from the results presented by other authors [13, 14]. Ultimately, the choice of treatment method is a joint decision of the oncologist and the patient, taking into consideration the medical indications, the effects of therapy on the basic parameters of the treatment effectiveness assessment, including the quality of life and preferences of the patient. In the age of anti-HER2 therapy availability and its effectiveness, the HER2 positive receptor patients are routinely treated with such therapy. The remaining patients are qualified for chemotherapy or hormone therapy, which in many cases have comparable results of treatment. Therefore, taking the QOL parameters into consideration may be crucial for such patients. In the analyzed material there were no formal limitations related to the performance status, comorbidities and other restrictions usually set in clinical trials. The decision to initiate treatment was made by clinical oncology specialists, included in this group were 101 (29%) patients aged over 70 years, of which 10 (3%) over 80 years of age. Notwithstanding, the presented analysis indicates that the results obtained in everyday clinical practice may be different in comparison to the results achieved in clinical trials, involving carefully selected groups of patients. In the presented material, serious adverse reactions (WHO 3rd and 4th degree) occurred in 40% of patients. In 23% of patients, at least two serious toxic reactions were noted. These results differ significantly from those obtained in large clinical trials and may additionally affect the QOL assessment.

Greater toxicity of chemotherapy in comparison with hormone therapy is understandable and in accordance with literature data. The presented data and its interpretation confirm the authors` hypothesis that treatment toxicity observed in daily clinical practice may be greater than that demonstrated in clinical trials and may negatively impact the QOL.

In the group studied, the patients undergoing hormone therapy were characterized by better cognitive functioning (less difficulty with memory and focus) and reported less severe systemic side effects of treatment than women treated with chemotherapy. However, there was no significant correlation between the cognitive performance of the studied women (QLQ-C30 functional scale) and the prospects assessment reported in the QLQ-BR23 questionnaire. In the authors` study no impact of demographic variables on the QOL of the surveyed women was demonstrated.

Other authors observed that women treated with mastectomy generally experience financial problems more often than women treated with breast conserving surgery. [15] The authors of this study noted a similar relationship among patients treated with chemotherapy in relation to those treated with hormone therapy. The character of the relationship between the perception of the general quality of life and the financial situation may suggest that a better financial situation promotes a neutral or positive overall QOL assessment, and a poor financial situation relates to a decidedly negative assessment. Perhaps this is due to the fact that a better financial situation as an additional psychological resource, shapes a more positive assessment of the health situation. Or maybe just the necessity of commuting to receive treatment, performing more medical procedures related to chemotherapy than with hormone treatment directly affects the financial situation, limiting the possibilities of professional work. An additional argument is that symptoms such as hair loss, nausea and vomiting may render working difficult. This observation can be considered in the context that chemotherapy significantly limits women’s involvement in work (only about 16% of women remain professionally active during treatment), which may reduce their ability to earn a living.

Arora et al., having examined the quality of life after surgical and systemic treatment, observed that sexual functioning was significantly worse in the group of women undergoing systemic treatment compared to patients treated only surgically [16]. The authors` research demonstrates similar conclusions but in the context of chemotherapy in relation to hormone therapy.

Researchers of quality of life of patients treated radically generally notice a higher quality of life of young women treated surgically for breast cancer and that older patients achieve better results on the scales describing physical, emotional, cognitive and social performance than younger women, and the future prospects (prognosis) are perceived more positively by older women [17, 18]. In contrast to the above observations, the authors` study did not show a correlation of demographic variables, including the age of the studied women, with the functional components of the quality of life of the studied women.

As expected, patients undergoing hormone therapy compared with patients receiving chemotherapy had better indicators of cognitive functioning, which was manifested by less memory and focus problems, and lower severity of systemic side effects. On the other hand, cognitive function gradually deteriorated when combining hormone therapy with intravenous infusions of vitamin C. Patients receiving vitamin C also had significantly worse appetite, increased diarrhoea, worse cognitive functions and greater financial problems. The reason for the first two phenomena is unclear, while the financial aspect may result from the high costs of this procedure.

The results of the authors` study are largely consistent with the above observations. In the multiple regression model, the authors` demonstrated that there is a positive correlation between better emotional functioning (lower psychological tension, lack of tendency to worry, lack of irritability, better mood) in women after treatment and assessment of future prospects in the study group. The better emotional functioning during the 6 months after the therapy, the better the assessment of future prospects.

The authors` believe that the data provided, as well as the results of other cited studies, indicate the validity of continuing research into the quality of life of palliative breast cancer patients in everyday clinical practice. The essence of the authors` research is that in the face of ever-changing methods and recommendations regarding palliative systemic treatment it is also essential to update psychological data necessary to effectively improve emotional functioning, general health and reduce anxiety in the women treated. The authors also point out the necessity to assess the influence of factors other than treatment on the quality of life of advanced breast cancer patients treated palliative.

## Conclusions

The presented results indicate the lack of influence of chemotherapy on quality of life of patients treated due to advanced, metastatic breast cancer, but hormonotherapy and trastuzumab therapy improved the quality of life of the treated patients in clinical practice. Women undergoing hormone therapy demonstrated better cognitive performance and at the same time reported less severe systemic side effects of treatment than women treated with chemotherapy. Additionally, in multiple regression analysis, a significant positive correlation between the “emotional performance” variable and the future prospects assessment was demonstrated. However, this presupposes some caution in extrapolating the results found in the literature to everyday practice and indicates the usefulness of population studies in assessing the effectiveness of new therapies, in large groups of patients in clinical practice.

## Data Availability

Please contact author for data requests.

## Abbreviation

QLQ: Quality of life
